# Clozapine (and norclozapine) concentrations are correlated with functioning, disability and side-effects when patients are treated with clozapine without the availability of therapeutic drug monitoring: A study of treatment-resistant schizophrenia inpatients

**DOI:** 10.3892/br.2025.2087

**Published:** 2025-11-18

**Authors:** Veroljub Petrovic, Dan Cohen, Dragic Bankovic, Branimir Radmanovic, Dragana Ignjatovic Ristic

**Affiliations:** 1 Special Hospital for Psychiatric Disorders Kovin, 26220 Kovin, Serbia; 2 Esdege Reigersdaal. J. Oudweg 2, 1703 DE Heerhugowaard, The Netherlands; 3 Faculty of Science, University of Kragujevac, 34000 Kragujevac, Serbia; 4 Faculty of Medical Sciences University of Kragujevac, 34000 Kragujevac, Serbia; 5 Clinic for Psychiatry, Clinical Centre of Kragujevac, 34000 Kragujevac, Serbia

**Keywords:** treatment-resistant schizophrenia, clozapine, side effects, dried blood stains, disability levels

## Abstract

In numerous countries, including Serbia, therapeutic drug monitoring (TDM) for clozapine is unavailable, leaving dose adjustments based solely on clinical judgment, could increase risk of therapeutic failure or adverse outcomes. The present study aimed to assess associations between clozapine and norclozapine serum concentrations, measured via Dried Blood Spot (DBS), and symptom severity, functioning, disability, and side effects in Serbian inpatients with treatment-resistant schizophrenia (TRS). In a cross-sectional design, patients with TRS with ongoing clozapine treatment and who did not meet exclusion criteria, were to be included. For clinical assessments of symptom severity, functionality, disability and side effects, were used: Simplified Interview for Negative and Positive Symptoms (SNAPSI), Clinical Global Impression (CGI), Global Assessment of Functioning (GAF), WHO Disability Assessment Schedule (WHODAS) and the Clozapine Adverse Effects Scale (GASS-C). Spearman's analysis showed no significant correlation between GASS-C scores and clozapine dose or plasma levels. WHODAS scores showed significant positive correlations with clozapine dose (ρ=0.409, P=0.006), clozapine (ρ=0.333, P=0.038) and norclozapine (ρ=0.481, P=0.002) levels. GAF scores negatively correlated with clozapine (ρ=-0.354, P=0.027) and norclozapine (ρ=-0.370, P=0.020). GASS-C scores were significantly associated with female sex (P=0.003), body mass index (BMI; ρ=-0.678, P<0.0005) and WHODAS scores (ρ=0.372, P=0.014). Regression analysis identified clozapine dose, clozapine level, BMI, SNAPSI and CGI scores as significantly associated with GASS-C (P=0.045). Norclozapine, BMI and CGI scores significantly associated with WHODAS (P=0.018). In the present study, it was found that clozapine and norclozapine concentrations are significantly associated with functioning, disability and side effects in patients with TRS treated without TDM. DBS-based monitoring may offer a valuable alternative for optimizing treatment. Further longitudinal studies are needed to clarify the clinical utility of (nor) clozapine level-guided care in such settings.

## Introduction

Although clozapine remains the only evidence-based pharmacological treatment for treatment-resistant schizophrenia (TRS) (1-4), decades of debate regarding potentially fatal side effects, especially agranulocytosis, continue to generate hesitation among clinicians and have not resulted in increased clozapine utilization, despite accumulating supportive evidence (4-6). TRS represents a major clinical challenge, and clozapine remains the gold standard for its management. Several factors, including sex, age, smoking habits and metabolic capacity, may influence clozapine and norclozapine serum concentration variability (1,7-10). The rationale for the present study stems from findings of the multicentric CLOSER study (11), which investigated clozapine plasma levels and outcomes in Serbian patients with treatment-resistant psychotic disorders treated without therapeutic drug monitoring (TDM). While that study provided valuable preliminary insights, it included patients from three different centers and covered a broader diagnostic spectrum, introducing institutional and demographic variability.

By contrast, the current study focuses on a localized and diagnostically homogeneous cohort of 43 patients with TRS, treated at the Special Hospital for Psychiatric Disorders ‘Kovin’ (SBPB Kovin), allowing for a more detailed evaluation of clozapine and norclozapine serum level variability, clinical scales and adverse effects. This targeted and context-specific design provides complementary evidence to the broader multicentric CLOSER dataset and enables more precise identification of factors contributing to interindividual pharmacokinetic variability and treatment outcomes in this specific population. Understanding these factors is essential for optimizing dosing strategies and improving therapeutic outcomes. Importantly, studies have shown that the occurrence of subjective side effects is not directly related to clozapine dosage (12-14). A significant but weak correlation has been observed between side effects and plasma clozapine concentrations (14). Seppälä *et al* (13) also reported an association between elevated norclozapine levels and symptoms of depression/anxiety, although causality could not be determined. Clozapine, particularly in TRS, has demonstrated superiority in improving quality of life and symptomatology, though data on its effects on functional disability remain limited (15,16).

In numerous countries, including Serbia, TDM for clozapine is not routinely available. This limitation contributes to underutilization of clozapine or, when it is used, to avoidance of higher therapeutic doses due to fear of adverse effects. Consequently, dosage adjustments are often based solely on clinical judgment, which may increase the risk of therapeutic failure or adverse outcomes (17).

Patients on clozapine require regular clinical monitoring obligation, including physical examinations and laboratory testing, to minimize the risk of side effects (18,19). Monitoring plasma concentrations of clozapine and its metabolite norclozapine enhances treatment safety and efficacy, supports adherence, and reduces the risk of toxicity (20). The timely detection of numerous previously overlooked side-effects has significantly improved wherever after clinicians implemented the Glasgow scale for clozapine side effects scale (12), that has been well validated and translated into Serbian (21). Clozapine is metabolized into N-desmethylclozapine (norclozapine) primarily by cytochrome P450 1A2, with lesser involvement of CYP3A4, 2D6, 2C9 and 2C19(7).

Schizophrenia, a disorder that affects cognition, affect, perception and thought content, is one of the leading causes of long-term disability worldwide (22). Up to 80% of patients experience functional impairments across multiple life domains (23).

The aim of the present study is to assess the association between serum concentrations of clozapine and norclozapine-measured with the Dried Blood Spot (DBS) method - and symptom severity, functionality, disability and side effects in Serbian inpatients with TRS.

## Materials and methods

### Study design and participants

The present cross-sectional study is part of a larger multi-center investigation, with previously published preliminary findings involving patients with schizophrenia and schizoaffective disorder focusing on relationship between clozapine levels and outcomes and clozapine level above the laboratory-alert level (11). Given the controversies of the nosological entity of schizoaffective disorder (24), and that current clinical recommendations are based on data from large trials in schizophrenia or extrapolations from samples of bipolar or schizophrenic patients (25). In the present study, focus was addressed exclusively on data from 43 adult patients diagnosed with TRS, treated at the SBPB Kovin, Serbia. All participants had been receiving a stable clozapine dose for at least three months, either as inpatients or outpatients. Written informed consent was obtained in line with the Declaration of Helsinki and Good Clinical Practice guidelines, following ethical approval by the SBPB Kovin Ethics Committee (approval no. 01-3615/3-3659/1; dated 29.11.2017; Kovin, Serbia).

### Inclusion and exclusion criteria

Inclusion criteria were: (i) Diagnosis of schizophrenia (ICD-10 codes F20.0-F20.9); (ii) age: 18-55 years; (iii) documented treatment resistance (non-response to at least two different antipsychotics) (26); and (iv) ongoing clozapine therapy. Exclusion criteria included: (i) Psychotic disorders outside the schizophrenia spectrum; (ii) bipolar or unipolar mood disorders; (iii) organic psychoses and (iv) substance-induced psychotic disorders.

### Demographic data

Demographic information included: Sex, age, Body Mass Index (BMI), obesity, education level, employment status, marital status, coffee and energy drink consumption.

### Clozapine therapy profile

Information was collected on clozapine dosage (daily and number of doses), as well as clozapine and norclozapine concentrations.

### Clinical data

Clinical history data included the place of treatment and number of psychiatric hospitalizations. The following standardized clinical instruments were used: GASS-C (Clozapine Adverse Effects Scale, Serbian version): Evaluates the frequency and distress of 16 common clozapine-related side effects on a 0-3 Likert scale over the past 7 days. Severity levels are categorized as mild, 0-16; moderate, 17-32; and severe, 33-48. Internal consistency: Cronbach's α=0.82(21).

*WHODAS 2.0 (Disability Assessment Scale)*: Assesses disability across multiple domains (27,28). Scores range from 1 (no difficulty) to 5 (extreme difficulty).

*Clinical Global Impression-Severity (CGI-S):* Clinician-rated global measure of illness severity on a 7-point scale (29).

*Global Assessment of Functioning (GAF)*: Evaluates social, occupational, and psychological functioning, ranging from 1 (severe impairment) to 100 (optimal functioning) (30,31).

*Simplified Interview for Positive and Negative Symptoms (SNAPSI)*: A structured interview tool designed to extract ratings from Brief Psychiatric Rating Scale (29), PANSS-6, and other scales, including integration of collateral information (32-35). All assessments were performed by a single psychiatrist with extensive experience using the aforementioned instruments. Only validated Serbian-language versions of all scales were used.

### Clozapine and norclozapine level determination

Clozapine and norclozapine concentrations were measured using the DBS method, which is cost-effective and suitable for resource-limited settings. DBS has been validated for clozapine, making TDM of clozapine accessible in parts of the world where it is currently unavailable. Single-layer filter paper cards (Whatman 903) were used, with correction factors applied to account for cellular components. DBS samples were obtained from venous blood collected at SBPB Kovin, following standard sampling procedures (36-38).

### Statistical analysis

Statistical analyses were conducted using SPSS v19 (IBM Corp.). Descriptive statistics were used for all variables. Group comparisons were performed using independent-samples t-tests or Mann-Whitney U-tests for two groups, and one-way ANOVA one-way or Kruskal-Wallis tests for three or more groups. Associations between categorical variables were analysed using Chi-square or Fisher's exact tests. Correlations between numerical variables were evaluated using Spearman's correlation coefficient ρ. Multivariate linear regression was used to determine predictors of GASS-C and WHODAS scores. P<0.05 was considered to indicate a statistically significant difference.

## Results

The demographic and clinical characteristics of the 43 patients with TRS treated with clozapine are included in Table I. Our sample primarily consisted of middle-aged patients, with a predominance of males (76.7%) over females (23.3%), and a long duration of illness and hospitalizations. This 3:1 male-to-female ratio reflects the local institutional population and may also align with meta-analytic evidence indicating a higher likelihood of TRS in men (39). Sex differences, including smoking habits and metabolic profiles, are relevant for interpreting clozapine and norclozapine concentrations (1,7-10,15). Most patients were smokers, with a mean BMI of 26.28±5. These characteristics are consistent with previously described TRS populations.

The association between clozapine side effects (assessed by GASS-C) and demographic, clinical characteristics and clozapine treatment profile are demonstrated in Table II. Patients with only one hospitalization tended to have higher levels of side effects compared with those with multiple hospitalizations, although this difference was not statistically significant (P=0.055). Notably, higher body mass and BMI were significantly associated with fewer side effects (GASS-C scores) (ρ=-0.613, P<0.0005; ρ=-0.678, P<0.0005, respectively, where ρ is Spearman's correlation coefficient and P is probability). Conversely, higher disability levels (WHODAS scores) correlated positively with the severity of side effects (ρ=0.372, P=0.014).

Correlations between clinical characteristics and clozapine therapy parameters are shown in Table III. A higher daily dose of clozapine was significantly correlated with higher plasma clozapine and norclozapine concentrations (ρ=0.515, P<0.001; ρ=0.647, P<0.001, respectively). Additionally, clozapine and norclozapine concentrations were strongly positively correlated (ρ=0.850, P<0.0005).

Multiple linear regression analysis identified that clozapine dose, clozapine concentration, BMI, SNAPSI scores and CGI-S scores are significantly associated with clozapine side effects (GASS-C). Norclozapine dose, BMI and CGI-S are significantly associated with disability (WHODAS).

The following model is given in Table IV. GASS-C=8.245-0.005·(Clozapine dose) + 0.010·(Clozapine concentration) -0.397·BMI + 0.769·SNAPSI -2.130·(CGI-S). Wilcoxon signed ranks test indicated no significant difference between observed and predicted GASS-C scores (P=0.441). The following model is also provided in Table IV. WHODAS=-16.963 + 0.027· (Norclozapine concentration) -0.652·BMI + 8.864·(CGI-S). No significant difference was found between observed and predicted WHODAS scores (P=0.837).

## Discussion

The present data indicated that female patients experience more clozapine adverse effects than males, and that lower BMI is associated with more side effects. Additionally, patients with fewer hospitalizations and higher disability (WHODAS scores) tend to report more side effects. Clozapine dose, plasma clozapine concentration, BMI, symptom severity (SNAPSI) and illness severity (CGI-S) were significantly associated with adverse effects, while norclozapine concentration, BMI and CGI-S were significantly associated with disability.

Despite the efficacy of clozapine in TRS and its role in suicide prevention among patients with schizophrenia, its use remains limited, with only 5-20% of eligible patients receiving clozapine therapy or continuing treatment in the long term (40-46). One possible explanation for this underutilization is the occurrence of adverse effects (for example, agranulocytosis and metabolic side effects), which negatively affect treatment adherence. Therefore, effective treatment strategies are necessary to facilitate long-term clozapine tolerance and improve its utilization patterns (47,48).

The patient sample in the present study aligns with the profile of patients with TRS commonly described in the literature, predominantly middle-aged males, often smokers, with low educational attainment and long illness duration (49-51). The observed male predominance in our cohort (3:1) is specific to the SBPB Kovin institutional population and may not reflect broader population trends in TRS. Nonetheless, a recent meta-analysis indicates that men are more likely than women to develop TRS (OR ≈ 1.57) (39). Sex differences could influence metabolic profiles, side-effect susceptibility, and smoking patterns, which are important factors to consider when interpreting drug concentrations and clinical outcomes (1,7-10).

Obesity and increased BMI are among the most frequently studied adverse effects of clozapine treatment (52,53). Some authors suggest that unhealthy dietary habits and low levels of physical activity, commonly observed among patients with schizophrenia, may also contribute to obesity (53,54). Furthermore, previous studies indicate that, compared with men, women on clozapine therapy tend to have higher BMI and glucose levels, a greater prevalence of triglyceride and LDL cholesterol dysregulation, and lower blood pressure values (55). These differences may be explained by findings showing that plasma concentrations of clozapine are generally higher in women than in men (56,57). Accordingly, the results of the present study also demonstrated that women report adverse effects more frequently than men (56).

Patients who were hospitalized for the first time reported more adverse effects, likely reflecting the initial adaptation to treatment. This finding is consistent with previous observations suggesting that adverse effects may decrease with continued treatment, thereby improving adherence (58).

The relationship between plasma clozapine levels and adverse effects is complex. The present study did not find a direct correlation between clozapine dose or plasma levels and adverse effects; however, multivariate regression analysis showed that both dose and plasma concentration were significantly associated when combined with other factors such as BMI and symptom severity. This highlights the individual variability in sensitivity to adverse effects within the therapeutic range. In the present study, higher disability levels (measured by WHODAS) were correlated with higher GASS scores, indicating more severe adverse effects. Similar findings have been reported in previous research. For example, a study by Gurcan *et al* (16), which assessed clozapine-related adverse effects, found a statistically significant correlation between the severity of adverse effects and increased levels of disability as measured by WHODAS (16). Greater disability (WHODAS) was associated with more severe adverse effects and illness severity (CGI-S), confirming that both contribute to the overall disability burden in TRS.

Additionally, norclozapine levels were also significantly associated with disability, supporting existing evidence that plasma norclozapine concentrations are associated with metabolic adverse effects that influence functionality. Although the exact mechanisms remain unclear, most studies show a stronger correlation between serum norclozapine levels and metabolic syndrome parameters than with serum clozapine levels (59-62).

Previous research also suggests a strong relationship between illness severity and disability, with illness severity serving as a predictor of disability level (63). An Indian study investigating the long-term impact of clozapine on disability and disease course, among patients treated with clozapine for an average of 5 years, found that higher CGI-S scores at follow-up, lower global improvement (CGI-I), and poorer efficacy index (CGI-E) were associated with greater disability (64). These findings are consistent with the current results, which also indicate that CGI-S scores have significantly association with disability. In addition to metabolic and environmental factors, genetic variability, including polymorphisms in enzymes such as CYP1A2 and CYP2D6, can significantly influence clozapine and norclozapine levels as well as treatment response (8-10,65). Another important aspect not addressed in the present study involves pharmacodynamic factors. Receptor characteristics and pharmacodynamic biomarkers may provide deeper insights into therapeutic response and will be included, together with genetic analyses, in the next phase of our multicentric research project (4,8,10,43). These results complement findings from the broader multicentric CLOSER study, which included diagnostically heterogeneous patients recruited across three centers. By focusing on a localized and diagnostically homogeneous cohort, the present study allows for a more detailed evaluation of interindividual variability in clozapine and norclozapine levels and associated clinical outcomes. This center-specific analysis contributes nuanced information that may be masked in larger heterogeneous samples.

The strength of the present study lies in its provision of real-world data on clozapine levels, effects, functioning, and side effects during titration in patients with schizophrenia. Relatively detailed clinical data were collected on patients treated with clozapine, and clinical outcomes were assessed using well-established and validated instruments.

However, the study has several limitations. First, the relatively small sample size is a limitation, even though validated and widely used instruments were employed, and clinicians were either trained in their use or had prior experience with them. The male predominance in our sample reflects both the institutional population and meta-analytic evidence that men are more likely to develop TRS (39), which should be considered when interpreting outcomes. Second, GASS-C is considered the gold standard for this purpose, it primarily measures the subjective burden of side effects as experienced by patients, which may not always align with objectively measurable or clinically verifiable adverse events. Third, although multivariate linear regression was used to identify independent predictors of psychiatrists' attitudes, the cross-sectional nature of the study prevents causal inferences between variables.

## Figures and Tables

**Table I:** Demographic, clinical characteristics and profile of clozapine therapy in patients with treatment-resistant schizophrenia.

Demographic characteristics	Mean ± SD or N (%)
Sex	
Male	33 (76.7%)
Female	10 (23.3%)
Age, years	51.35±10.11
Obesity	
Male	0.0%
Female	24.2%
Body mass index (kg/m^2^)	26.28±5.04
Coffee (daily)	2.79±1.08
Coke (weekly, in liters)	0.10±0.32
Cigarettes (daily)	17.67±12.12
Marital status	
Single	29 (67.4%)
Married	7 (16.3%)
Divorced/widowed	7 (16.3%)
Educational level	
No formal education	4 (9.3%)
Primary school	13 (30.2 %)
Vocational education	7 (16.3%)
High school 8-12	19 (44.2%)
College 12-16	3 (7.0%)
University	1 (2.3%)
Employment status	
Unemployed	21 (48.8%)
Pensioner	22 (51.2%)
Clinical characteristics	Mean ± SD or N (%)
Number of hospitalizations	
1	20 (46.5%)
2	23 (53.5%)
Place of treatment	
Hospital	36 (83.7%)
Health care center	7 (16.3%)
GASS-C	3.14±4.039
WHODAS	12.28±10.64
CGI-S	4.67±1.017
GAF	54±14.5
SNAPSI	19.07±5.351
Clozapine therapy profile	N (%) or median (25-th percentile - 75-th percentile)
Daily clozapine dose frequency	
1	4 (9.3%)
2	24 (55.8%)
3	15 (34.9%)
Clozapine dose (mg/d)	200.00 (150.00-300.00)
Clozapine concentration (ng/ml)	309.40 (147.09-507.93)
Norclozapine concentration (ng/ml)	133.39 (71.40-207.57)

BMI, Body Mass Index; GASS-C, Serbian version of the scale for evaluating clozapine side effects; WHODAS, Disability Assessment Scale; CGI-S, Clinical Global Impression Scale-Severity; GAF, Global Assessment of Functioning; SNAPSI, Simplified interview for negative and positive symptoms.

**Table II:** Association of adverse effects of clozapine with demographic, clinical characteristics and profile of clozapine therapy in patients with treatment-resistant schizophrenia.

	GASS-C			
Variables	N	Mean ± SD	t-test	P-value
Sex			4.434	0.003
Male	33	1.85±2.93		
Female	10	7.40±4.40		
Obesity			10.931	0.001
No	24	3.38±4.42		
Yes	8	0.00±0.00		
Number of hospitalizations			3.892	0.055
1	20	4.40±4.99		
2	23	2.04±2.64		
Number of clozapine doses			5.515	0.008
1		0.00±0.00		
2		2.17±3.18		
3		5.53±4.66		

GASS-C, Serbian version of the Clozapine Adverse Effects Assessment Scale.

**Table III:** Correlation of clinical characteristics and profile of clozapine therapy in patients with treatment-resistant schizophrenia.

Variables	Daily dose of clozapine (mg/d)	Clozapine concentration (ng/ml)	Norclozapine concentration (ng/ml)
GASS-C	ρ=0.137 P=0.382	ρ=0.098 P=0.554	ρ=0.133 P=0.420
WHODAS	ρ=0.409 P=0.006	ρ=0.333 P=0.038	ρ=0.481 P=0.002
CGI-S	ρ=0.368 P=0.015	ρ=0.202 P=0.218	ρ=0.236 P=0.148
GAF	ρ=0.249 P=0.108	ρ=-0.354 P=0.027	ρ=-0.370 P=0.020
SNAPSI	ρ=0.209 P=0.508	ρ=0.198 P=0.538	ρ=0.346 P=0.271

GASS-C, Serbian version of the Clozapine Adverse Effects Assessment Scale; WHODAS, Disability Assessment Scale; CGI-S, Clinical Global Impression Scale-severity; GAF, Global Assessment of Functioning; SNAPSI, Simplified interview for negative and positive symptoms; ρ, Spearman's correlation coefficient; P, probability.

**Table IV:** Results of multiple linear regression analysis, where clozapine-related adverse effects and disability in patients with treatment-resistant schizophrenia are dependent variables.

A, Dependent variable GASS-C	B	P-value
Constant	8.245	
Clozapine dose (mg/d)	-0.005	0.045
Clozapine concentration (ng/ml)	0.010	0.009
BMI (kg/m^2^)	-0.397	0.010
SNAPSI	0.769	0.012
CGI-S	-2.130	0.047
B, Dependent variable WHODAS	B	P-value
Constant	-16.963	
Norclozapine concentration (ng/ml)	0.027	0.018
BMI (kg/m^2^)	-0.652	0.013
CGI-S	8.864	<0.0005

GASS-C, Serbian version of the Clozapine Adverse Effects Assessment Scale; BMI, body mass index; SNAPSI, Simplified interview for negative and positive symptoms; CGI-S, Clinical Global Impression Scale-severity; WHODAS, Disability Assessment Scale; B, coefficient of multiple linear regression.

## Data Availability

The data generated in the present study may be requested from the corresponding author.
